# Simulation models for training skin flap and graft surgery in the head region: a narrative review

**DOI:** 10.1097/j.pbj.0000000000000299

**Published:** 2025-09-10

**Authors:** Margarida Nogueira, Abel Nicolau, Cristina Granja

**Affiliations:** a Faculty of Engineering, University of Porto, Porto, Portugal; b RISE-Health, Faculty of Medicine, University of Porto, Porto, Portugal

**Keywords:** dermatology, head models, head surgery, simulation, surgical training

## Abstract

**Background:**

The demand for skill development in the surgical field is critical to achieve the best functional and aesthetic patient results. Therefore, the use of simulation models has become necessary and integrated in training, holding substantial importance in skin reconstruction in the head. These models, while varying for realism and training applications, offer a low-stress, safe, and replicable environment for interns and residents to enhance their surgical technique.

**Objective:**

The aim of this review was to analyze the various head simulators designed for skin flap and skin graft training. The anatomical representation, selected materials, manufacturing process, and validation approaches were examined to provide an overview of the characteristics of the models. In addition, their impact on training was assessed, categorizing the outcome as positive, negative, or with no effect, based on the findings of the reviewed studies.

**Methods:**

Published literature on PubMed and Scopus was gathered through relevant keywords and phrases related to dermatological facial surgery simulators. A narrative synthesis was conducted based on the reporting guidelines of the synthesis without meta-analysis method. The head models were evaluated with overall performance as the primary outcome and confidence, planning and design, and execution as the secondary outcomes.

**Results:**

Thirteen studies on head models for skin procedures were identified between 2004 and 2023. All the simulators reviewed demonstrated variability for supported techniques, composition, manufacturing methods, anatomical detail, and validation approach. Eleven studies demonstrated that the models improved at least one of the selected outcomes. No model was targeted for skin graft reconstruction. Furthermore, none of the models integrated objective feedback mechanisms.

**Conclusions:**

Simulation was proved to enhance the surgical training of dermatological reconstructions in the head, despite variations in realism, complexity and production process. Future efforts should prioritize higher anatomical accuracy, cost-efficiency, and integration of feedback mechanisms to improve the educational value of these tools.

## Introduction

### Challenges in surgical training

The landscape of surgical training faced growing obstacles in recent years. Factors including scarcity of procedures, rise of subspecializations, centralization of surgical services, and patient safety concerns have played a role in limiting hands-on training opportunities.^1,2^ These aspects underlined the need to create practical tools to reinforce learning. It is the training during residency that provides the necessary technical skills to achieve the best functional and aesthetic patient results.^3^

### Need for specialized training

There has been an increase in cases of the various forms of skin cancer, with the nonmelanoma skin cancer ranking as the most prevalent malignancy in the western world.^4^ The head and facial region are the most affected, accounting for more than 80% of lesions in the head and neck region.^5^ In many instances, the solution involves the surgical removal of the cancerous cells, highlighting the importance of mastering surgical techniques.^6^

These removal procedures, particularly skin flaps and grafts, stand out because of their complexity and delicacy, with flaps often presenting the greatest difficulty. While a skin graft involves completely transferring a portion of skin from one region to another, a skin flap remains attached to the donor site.^7^ Complications in these surgeries encompass face tension, ischemia, hematologic and infection issues, and even necrosis because of the internal structures and dynamics of blood flow in the face. To mitigate these risks, it is vital for tissues to be manipulated correctly. Besides, opting for the most suitable method and preplanning precisely are crucial to a successful result, underlining the importance of a comprehensive assessment of the defects.^8,9^

A survey conducted between 2013 and 2014 to 42 directors of dermatology residency programs in the United States revealed that residents acting as the lead surgeon in these types of reconstructions are only 52%.^10^ Gaining enough practice to build proficiency in these procedures is complicated, and expertise is particularly relevant in head surgeries, where maintaining both visual harmony and functional integrity is crucial.^8,11^

### Simulation in surgical education

Surgical interns and residents traditionally learn through a combination of theoretical classes, hands-on practice with skin pads and animal models, and direct interaction with patients.^10^ When compared with conventional methods, simulation provides a safe, low stress environment; facilitates the acquisition of technical skills; and reduces time of the performance and intraoperative errors.^12^ Ultimately, it improves patient outcomes. In fact, studies in dermatologic surgery simulation have garnered positive feedback: a survey from 2013 showed 75% of dermatology residents agreed that simulators are beneficial for acquiring, assessing, and reinforcing procedural skills and 91% supported that simulation should be a mandatory component of residency.^12^

Regarding skin flaps and grafts, realistic simulation models become crucial. Expertise involves manipulation of tissues, 3D conceptualization, and mastering techniques of various options for reconstructions. Experiential learning is key to understanding what can influence flap and graft design, which is best developed through hands-on practice.^13^

## Methodology

Published literature on MEDLINE (PubMed) and Scopus was gathered through relevant keywords related to dermatological head surgery simulations, namely, head, face, skin, flap, graft, training, surgery, simulation, model, and dermatology. These keywords were combined using Boolean operators (AND, OR). Zotero was used for reference management and duplicate removal and a single reviewer conducted the studies' screening. The selection process was documented using the preferred reporting items for systematic reviews and meta-analyses (PRISMA) flow diagram. To include updated articles and avoid unreliable comparisons, only studies published after 2000 were considered. Data were displayed in tables, with the studies ordered chronologically.

To improve the quality of the review, a narrative synthesis was performed based on the reporting guidelines of the synthesis without meta-analysis (SWiM) method. The head models were analyzed according to a set of key features, including skills trained, anatomical representation, materials selected, manufacturing methods, and validation approaches, to facilitate a comparison and assessment of their role in surgical training. The primary outcome assessed was overall performance, while the secondary outcomes included confidence, planning and design, and execution, as these parameters were the most frequently evaluated. The analysis approach was based on the direction of effect, since the studies did not present common quantitative data and were considered as favoring the intervention if at least one of the outcomes was improved. Vote counting based on the direction of effect was used to assess if research provided evidence of the intervention's effectiveness.

## Results

### Search results

**Figure 1.**
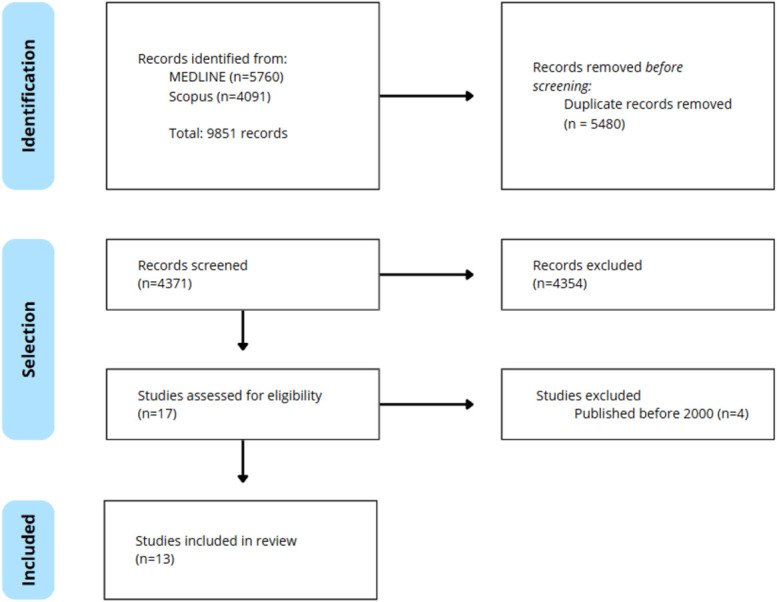
PRISMA (preferred reporting items for systematic reviews and meta-analyses) flow diagram of the selection process.

The PRISMA flow diagram, in Figure 1, presents the studies' selection process. The initial search retrieved 9851 records, 5480 of which were duplicates. After screening titles and abstracts, 4354 of the remaining 4371 studies were excluded. The remaining 17 publications underwent a full-text review, leading to the inclusion of 13 studies.

### Characteristics of head models

For the simulation of head surgeries, models that replicate both the characteristics of human skin and the underlying facial structure provide more accurate training. Reconstruction in this region can be particularly challenging because of the intricate geometry in certain designs and techniques. This underlines the importance of realistic head models, which offer invaluable practice opportunities, better preparing trainees for these types of procedures.^14^

The following table (Table 1) presents a comparative overview of research in simulation head models for teaching skin flaps. It addresses a range of simulators developed between 2004 and 2023, with detailed insights into various aspects of their design and functionality.

**Table 1:** Summary of head models for surgery simulation and training approaches.

Study	Sample size	Skills trained	Anatomical representation	Materials selected	Manufacturing method	Validation approach
Liew et al (2004)^15^	20 participants	Z-plasty and rhomboid flaps	Skin (epidermis, dermis, hypodermis), simulated lesions and skull	Soft silicone rubber for hypoderm is and silicone for top layers of the skin	Negative molding of face and casting in acrylic base; wax carving for facial structure	Workshop session with didactic lectures before practice on the simulator
Nicolaou et al (2006)^16^	Not mentioned	Local flaps, like rhomboid or bilobed flap	Skin (as one layer), simulated lesions, head	Pins for lesion simulation, cling film for skin layer, styrene mannequin head	Styrene mannequin covered with cling film	Practice on the simulator, where trainees were guided through drawing a local flap design
Chan and Dalal (2009)^17^	Not mentioned	Not mentioned	Skin (epidermis and dermis) and facial mold	Chicken thigh skin to simulate human skin, plastic mask covered in cling film for facial mold	Assembly of mask and cling film, followed by the placement of the chicken skin	Not mentioned
Chipp and Rayatt (2011)^18^	20 participants	Forehead flap and local flaps for lip reconstruction	Periosteum, muscle, dermis, epidermis and facial mold	Mefix (periosteum), Microfoam (muscle), Allevyn Adhesive (skin layers), plaster for facial model	Negative molding of face with alginate, cast in plaster, layered with adhesive dressings to simulate tissues	Postpractice questionnaire
Voigt et al (2012)^19^	Not mentioned	Variety of suture techniques dermatoplasty and skin flaps	Skin (epidermis and dermis), simulated lesions, and facial mold	Silicone for the skin, plaster powder for facial structure	3D printing of facial mold with plaster through CT data and silicone coated	Workshops for beginners and expert surgeons, with increasing complexity of exercises. An evaluation sheet was completed to evaluate the simulator
Davis et al (2013)^4^	Not mentioned	Rhomboid flap	Skin (as one layer) and skull	Medium- density foam (5 mm) for skin and human skull	Foam attached to skull with Velcro pads	Trainees practiced on the model
Hassan et al (2014)^20^	Not mentioned	Transposition and rotation flaps. Rhomboid, square peg in a round hole, forehead, glabella, bilobed, nasolabial, hatchet, staggered ellipse, Abbe, Estlander, and McGregor cheek flap	Skin (epidermis and dermis) and head	Porcine skin to simulate human skin, mannequin head and pins to secure the assembly	Mannequin head draped with two fragments of porcine skin, held into place with pins at strategic points	Residents practiced on the model, before training on cadavers
Taylor et al (2015)^13,21^	10 participants	Z-plasty, bilobed, rhomboid, and paramedian forehead flap	Skin (as one layer), facial mold and head	Gelatin:sorbitol-water:glycerin with the ratio of 1:2.5:3 to simulate skin and plaster of Paris for the facial mold	Negative molding using plaster of Paris over an expanded polystyrene foam (EPSF) head covered in modelling clay, followed by casting the gelatin mix to simulate skin	Lecture before practicing on the simulator. An 18-question Likert survey assessed realism, educational value, and task effectiveness
Ueda et al (2017)^22^	6 participants	Local flaps (bilobed) and cheiloplasty	Skin (one layer for the outer skin and another for the subcutaneous tissue), facial mold. Two models: normal face and face featuring a cleft lip	Silicone for the subcutaneous tissue and polyurethane for the skin outer skin (1 mm). Salt granules used for the facial mold	3D printing of the face based on CT/MRI data, made from salt granules, polyurethane painted inside the mold (1 mm), followed by silicone pouring and curing	Trainees practiced on the model
Kite et al (2018)^23^	13 participants	Forehead, bilobed, and nasolabial flaps	Skin (including hypodermis) and facial mold	Foam base for the face, overlaid with multiple silicone layers to represent the skin	Assembly of the silicon layers on top of the facial mold	The session started with videos on local flaps, followed by training in suture pads and finishing on the head model. A survey was conducted after practicing on the model, evaluating the skin and tissue simulated and the effectiveness as a teaching model
Powell et al (2019)^24^	7 participants	Z-plasty, V–Y, rhombic flap, rectangular advancement, bilobe, O–T, pinwheel	Skin (two layers, bottom being hypodermis) and facial mold	Silicone for skin (3 mm) and subcutaneous layers (6 mm), PLA for facial mold	3D printing of the facial mold using CT scans and CAD software, followed by negative casting to pour and cure the silicone	Postpractice questionnaire on physical attributes, realism, performance, application, and improvement
Yang et al (2021)^25^	15 participants	O-T and rhombic flaps	Skin (outer layer with 3 mm and hypodermis with 6 mm), facial mold	Silicone for the skin layers. No further materials were mentioned	3D printed facial model. No further methods were described	Model previously validated by experienced surgeons. Participants received didactic lectures and were divided into a control group practicing on paper illustrations and a Simulator group using the model. Pre-exercise and postexercise Likert scale surveys were implemented to determine the trainees' knowledge and assess simulator's utility, realism and effectiveness
Shay et al (2023)^14^	18 participants	Bilobe flap for nasal defects	Skin with 3 mm and facial mold	PLA for the face, Smooth- ON Dragonskin 10 for skin, polyester mesh for reinforcement of nose region	CT-based design, 3D printing of the facial mold, silicone layer and polyester mesh application	No validation of the model was assessed

3D, 3-dimensional; CAD, computer-aided design; CT, computed tomography; MRI, magnetic resonance imaging; PLA, polylactic acid.

The simulators were used to evaluate numerous reconstructions. The skills assessed in the experiments include both basic techniques and more challenging procedures related to skin flaps. It is also worth mentioning that no model explored skin grafts.

Anatomical accuracy varied among the range of models. The skin, which is the most vital region, was seen with one to three layers, including epidermis, dermis, and hypodermis. Nine models used a facial mold, instead of the fully representation of the head for training. The skin and the face were consistently present across the training tools, but a fraction had additional features, such as muscle and simulated lesions, such as tumors.

The materials used were both synthetic and biological. Seven models selected silicone as the best representative of human skin, yet some models also opted for foam. Porcine and chicken skin were also used as an attempt to enhance realism. The manufacturing methods spanned from manual assemblies to 3D printing models, based on computed tomography (CT) or magnetic resonance imaging (MRI) scans.

Validation involved practical training in the simulator. Many studies did not assess formally the participant's feedback, opting for free-form approaches, while others conducted surveys to evaluate aspects such as realism, educational value, and utility. Some validations were more comprehensive, comprising prior lectures and exercises for assessment of performance and effectiveness of the model.

Another relevant aspect to take into consideration is the level of realism of the materials used in the different models. Some studies collected participants feedback on the topic. Silicone was praised for its similarity to human skin, yet limitations were also noted, as its ability to hold sutures was not as effective as desired.^19,24^ Moreover, it also revealed to be challenging for mimicking tissue mobility.^22^ Silicone was rated higher for realism for replicating fat tissue compared with skin.^24^ On the other hand, the gelatin model was found to retain its shape, failing to replicate tissue mobility and flexibility as well.^13,21^

Table 2 presents the primary and secondary outcomes reported across the studies assessed. Each study is analyzed based on its findings in these categories to indicate whether the intervention was favored, or if the outcome favored the control, showed no effect, or was inconclusive/non-applicable.

**Table 2:** Analysis of primary and secondary outcomes across the reviewed studies.

Study	Primary outcome	Secondary outcomes			
	Overall performance	Planning and design	Confidence	Execution	
Liew et al (2004)^15^	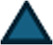	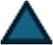	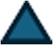	NA	Favors intervention
Nicolaou et al (2006)^16^	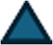	NA	NA	NA	Favors intervention
Chan and Dalal (2009)^17^	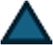	NA	NA	NA	Favors intervention
Chipp and Rayatt (2011)^18^	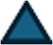	NA	NA	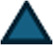	Favors intervention
Voigt et al (2012)^19^	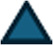	NA	NA	NA	Favors intervention
Davis et al (2013)^4^	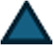	NA	NA	NA	Favors intervention
Hassan et al (2014)^20^	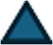	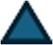	NA	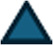	Favors intervention
Taylor et al (2015)^13,21^	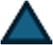	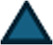	NA	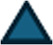	Favors intervention
Ueda et al (2017)^22^	IC	NA	NA	NA	IC
Kite et al (2018)^23^	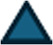	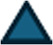	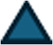	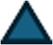	Favors intervention
Powell et al (2019)^24^	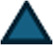	NA	NA	NA	Favors intervention
Yang et al (2021)^25^	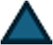	NA	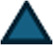	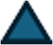	Favors intervention
Shay et al (2023)^14^	NA	NA	NA	NA	NA

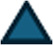
, Improvement; IC, inconclusive; NA, not applicable.

Ueda et al did not provide clear data on the effectiveness of the training, making it difficult to evaluate the model with the chosen parameters.^22^ Shay et al^14^ did not focus on the validation of the model. In several studies, it was not possible to analyze the secondary outcomes, as they were not specifically mentioned. Regarding the vote counting, 11 studies favored the intervention, 1 study was inconclusive, and 1 study did not apply these criteria in the validation. These results reflect the evidence trend that favors the intervention.

Owing to advancements in simulation and the rising need of surgical training, two products have already been commercialized, offering training opportunities for dermatology and head reconstructive surgery. Table 3 presents the available head models that aim to fill this educational gap.

**Table 3:** Summary of commercialized products of head models for surgery simulation.

Company	Model name	Key features	Applications
SimSkin^26^	Cosmo	Epidermis, dermis, and subcutaneous layers with realistic manipulation	Advanced suturing, flaps, and grafts
	Il Duomo	Epidermis, dermis, and subcutaneous layers with realistic manipulation Advanced features and internal anatomy	Complex and detailed reconstructions

A study conducted a performance validation of the Il Duomo Basic with Tumor. Participants rated the anatomy and reusability as the highest scoring parameters, but the texture of the skin lacked realism. While medical students stated that the simulator was too complex for learning basic skills, dermatology residents appreciated its value and declared its potential as a practicing tool.^27^

## Discussion

### Comparative analysis of head models

The most significant factor that differentiates head models is the level of anatomical accuracy. Models that comprise biological materials, such as chicken or porcine skin,^17,20^ have been used for their close similarity to human tissues. However, these materials are not as reliable when it comes to replicating intricate anatomy and movements around specific facial features (eyes, mouth, etc).

Silicone was the most frequent choice for skin representation. Thus, it is widely regarded as one of the best materials for creating skin surgical simulators, more specifically in the head and face. It can replicate the texture of human skin relatively well, providing a more realistic tactile experience when comparing with other materials.

Regarding the manufacturing process, newer models like the one developed by Powell et al^24^ offer significant improvements in realism. The use of 3D printing, combined with CT and MRI data, allows personalization and accurate anatomical features. High levels of satisfaction were reported in models' precision, indicating the potential to eventually replace cadaveric or animal-based simulations. Nevertheless, even these advanced tools faced limitations in mimicking the complexity and feel of tissue, particularly skin flexibility.

Some models have revealed to improve confidence, technical skills, and planning of procedures, while almost all proved to be effective in improving the overall performance of trainees. This demonstrates the essential role of hands-on experience in skill development, which theoretical or virtual simulations cannot fully replicate. It was shown that 11 studies favored the intervention, indicating a strong support for the effectiveness of head models.

A recurring issue in simulation is the balance between cost and realism. Some of the reviewed models present potentially inexpensive solutions but may not be the best options for the representation of advanced surgical scenarios, due to their simplicity. This is concerning for higher-level training, where anatomy and sensation are essential. On the other hand, 3D-printed models provide a more detailed and anatomically accurate representation of facial features but at a higher cost. Nonetheless, even the most expensive and advanced model, the Il Duomo, could not fully mimic the dynamic properties of human skin. The challenge is to guarantee that models are accessible, without compromising the quality of training.

### Identified gaps in head models

A notable gap in head models is the minimal focus on simulators built specifically for skin graft procedures, as only one commercialized model (Cosmo) served this purpose, but not exclusively.^26^ This imbalance may be because grafts are a less common approach and technically less demanding.^7^ Nonetheless, this overlooks the importance of simulating specific challenges for grafts, such as achieving proper settlement and fixation.

The most substantial gap identified is the lack of feedback mechanisms integrated in the models to objectively assess performance. While most simulators are acknowledged to improve surgical skills, the absence of evaluation tools prevents providing quantitative data on skill acquisition, such as force applied or tissue tension. Such metrics could be crucial for refining technique and recognizing procedures where interns and residents need reinforced practice. The addition of sensor-based systems and software capable of tracking these parameters could significantly increase the tools educational value.

Regarding the timing of feedback delivery, research by Shay et al^14^ concluded that feedback at any stage enhances long-term performance, as supported by psychological studies. Nonetheless, receiving feedback during the exercise leads to greater immediate improvement.

Therefore, including a feedback system would be valuable, enabling users to engage in autonomous practice and receive insights of their performance in real time. These mechanisms could detect mistakes, suggest improvements, and monitor progress, ultimately enriching the learning experience.

### Limitations

The studies included present limitations that hinder comparisons and the reliability of outcomes in the review. The lack of standardized methodologies, evaluation criteria, metrics, and reported findings limit an objective assessment of the subject. Research also focuses on short-term results, such as satisfaction or immediate skill improvement, without considering long-term benefits. In addition, the absence of control groups and small sample sizes also decreases the reliability of the results, while the vote counting based on direction of effect method lacks insight into the magnitude of the positive effects observed.

Relevant research may have been excluded from the review, due to limitations in database access, language barriers, or selection criteria. The SWiM reporting items were a valuable methodology to improve the review's quality and structure, yet the incomplete implementation of certain items, such as the assessment of certainty, may limit standardization and robustness of the findings.

## Conclusions

Simulation models could transform surgical training when it comes to skin procedures by providing a structured, hands-on learning environment. Head models are particularly beneficial for training skin flap and graft surgeries in the head region. They improve anatomical realism, offering a platform to practice techniques in controlled and reproducible conditions. As technology progresses, these models have the potential to comprise a comprehensive training experience. Integrating feedback systems would be a significant enhancement to enable autonomous and objective skill development.

Therefore, future directions should concentrate on head models with anatomical and sensorial realism, incorporating feedback mechanisms, and reducing production costs. These developments would allocate these simulators as viable and effective tools for surgical education in head and facial skin reconstruction.
